# Abnormal spatial diffusion of Ca^2+ ^in F508del-CFTR airway epithelial cells

**DOI:** 10.1186/1465-9921-9-70

**Published:** 2008-10-30

**Authors:** Fabrice Antigny, Caroline Norez, Anne Cantereau, Frédéric Becq, Clarisse Vandebrouck

**Affiliations:** 1 Institut de Physiologie et Biologie Cellulaires, Université de Poitiers, CNRS, 86022 Poitiers, France

## Abstract

**Background:**

In airway epithelial cells, calcium mobilization can be elicited by selective autocrine and/or paracrine activation of apical or basolateral membrane heterotrimeric G protein-coupled receptors linked to phospholipase C (PLC) stimulation, which generates inositol 1,4,5-trisphosphate (IP_3_) and 1,2-diacylglycerol (DAG) and induces Ca^2+ ^release from endoplasmic reticulum (ER) stores.

**Methods:**

In the present study, we monitored the cytosolic Ca^2+ ^transients using the UV light photolysis technique to uncage caged Ca^2+ ^or caged IP_3 _into the cytosol of loaded airway epithelial cells of cystic fibrosis (CF) and non-CF origin. We compared in these cells the types of Ca^2+ ^receptors present in the ER, and measured their Ca^2+ ^dependent activity before and after correction of F508del-CFTR abnormal trafficking either by low temperature or by the pharmacological corrector miglustat (N-butyldeoxynojirimycin).

**Results:**

We showed reduction of the inositol 1,4,5-trisphosphate receptors (IP_3_R) dependent-Ca^2+ ^response following both correcting treatments compared to uncorrected cells in such a way that Ca^2+ ^responses (CF+treatment *vs *wild-type cells) were normalized. This normalization of the Ca^2+ ^rate does not affect the activity of Ca^2+^-dependent chloride channel in miglustat-treated CF cells. Using two inhibitors of IP_3_R1, we observed a decrease of the implication of IP_3_R1 in the Ca^2+ ^response in CF corrected cells. We observed a similar Ca^2+ ^mobilization between CF-KM4 cells and CFTR-cDNA transfected CF cells (CF-KM4-reverted). When we restored the F508del-CFTR trafficking in CFTR-reverted cells, the specific IP_3_R activity was also reduced to a similar level as in non CF cells. At the structural level, the ER morphology of CF cells was highly condensed around the nucleus while in non CF cells or corrected CF cells the ER was extended at the totality of cell.

**Conclusion:**

These results suggest reversal of the IP_3_R dysfunction in F508del-CFTR epithelial cells by correction of the abnormal trafficking of F508del-CFTR in cystic fibrosis cells. Moreover, using CFTR cDNA-transfected CF cells, we demonstrated that abnormal increase of IP_3_R Ca^2+ ^release in CF human epithelial cells could be the consequence of F508del-CFTR retention in ER compartment.

## Introduction

The existence of distinct membrane localizations and multiple isoforms of inositol 1,4,5-trisphosphate (IP_3_) receptors (IP_3_R) within the same cell type may explain the complex spatiotemporal patterns of Ca^2+ ^release from IP_3_-sensitive calcium pools in epithelial cells. In addition to requiring IP_3_, IP_3_R are regulated in a biphasic manner by direct interaction with Ca^2+^, *i.e*. activation at low concentrations (up to 0.3 μM) and inhibition at higher concentrations (0.5–1 μM) [1]. The different modes of interaction of IP_3_R with Ca^2+ ^are involved in the complex feedback regulation of the Ca^2+^release [2]. IP_3_R activity is also regulated by Ca^2+^-independent accessory proteins, Mg^2+^, redox potential and ATP [3]. Furthermore, a local Ca^2+ ^discharge by photolysis of NP-EGTA technique can activate the IP_3_Rs Ca^2+ ^release. For example, the type 3 IP_3_R remaining open in the presence of high Ca^2+ ^concentration, initiates a rapid, large and almost total release of Ca^2+ ^from intracellular stores [4]. These properties place IP_3_Rs at the heart of calcium signalling pathways.

Recent studies have demonstrated higher intracellular Ca^2+ ^mobilization in Cystic Fibrosis (CF) compared to normal human nasal [5] or bronchial [6] epithelia. Cystic Fibrosis is the most frequent lethal autosomal recessive genetic disease in Caucasian population. The most common mutation in CF is a deletion of phenylalanine at position 508 in the Cystic Fibrosis Transmembrane conductance Regulator protein (F508del-CFTR). F508del-CFTR protein is misfolded, trapped in the endoplasmic reticulum (ER) by the ER quality control (ERQC) [7] and subsequently submitted to proteasomal degradation [8].

In this report we monitored the cytosolic Ca^2+ ^transients using the flash photolysis technique to uncage caged Ca^2+ ^into the cytosol of nitrophenyl-EGTA (NP-EGTA) loaded human CF nasal epithelial CF15 cells [9], human CF tracheal gland CF-KM4 cells [10] and human non-CF tracheal gland epithelial MM39 cells [11]. We also used the membrane-permeable UV light photolysis caged IP_3 _analogue (iso-Ins(1,4,5)P3/PM) to examine the consequence on the local IP_3_R Ca^2+ ^release of rescuing F508del-CFTR by the pharmacological corrector miglustat [12] and after culturing cells at low temperature [13].

## Materials and methods

### Cells

Human nasal epithelial JME/CF15 cells (F508del/F508del) were grown at 37°C in 5% CO_2 _under standard culture conditions [9]. Human CF and non-CF tracheal gland serous CF-KM4 and MM39 cells were cultured as previously described [5]. The CF-KM4 cells transducted with the lentiviral vector expressing the wild-type CFTR cDNA [14] (named in this study CF-KM4 reverted), were generously given by Dr. Christelle Coraux (INSERM U514, Reims University, IFR53, Reims, France).

### Extraction of IP_3_R mRNA and reverse transcription

Total RNA was extracted using RNABle^® ^(Eurobio), according to the protocol provided by the manufacturer and mRNA was reverse transcribed to cDNA as described elsewhere [15]. The specific oligonucleotide primers used for each subtype of the IP_3_Rs are presented Table 1. The temperature cycling conditions were initial melting at 94°C for 5 min, annealing at 56°C for 2 min followed by 30 cycles of 72°C for 30 s, 94°C for 30 s, annealing of 56°C for 30 s and a final extension at 72°C for 5 min.

**Table 1:** Specific primers for each IP_3_Rs subtype

	Accession number	Primer sens	Primer anti-sens	bp
hlITPR 1	NM_002222	5'-AACCGCTACTC TGCCCAAAA-3'	5'AGTTTGTTGAGTAGCACTGCGTCT-3'	86
hlITPR 2	NM_002223	5'-GCGATCTGCA CATCTATGCTG-3'	5'-AAGTATTAATGTA GGCCCAAGACCTATT-3'	117
hlITPR 3	NM_002224	5'-GGGCTCTCG GTGCCTGA-3'	5'-GGAGGGCTTGC GGAGAA-3'	150

### Quantification of IP_3_R mRNA by RT-PCR

Quantitative PCR was used to determine the copy numbers of IP_3_R1, IP_3_R2, and IP_3_R3 in mRNA extracted from CF15 cells in different conditions. The IP_3_R mRNA quantities were normalized against β-actin. Quantitative PCR were performed on the ABI Prism 7700. The specific oligonucleotide primer used for each subtype of the IP_3_Rs is presented Table 1. For β-actin-cDNA, the primers were 5'-TGTGGATCGGCGGCTC-3' and 5'-ACTCCTGCTTGCTGCTGATCCAT-3' (900 nM for each primer). The probe taqman FAM used was 5'FAM-TGGCCTCGCTGTCCACCTTCCA-TAMRA3' (200 nM). The temperature cycling conditions were: initial melting at 94°C for 5 min, annealing at 56°C for 2 min followed by 30 cycles of 72°C for 30 s, 94°C for 30 s, annealing of 56°C for 30 s and a final extension at 72°C for 30 s. Each sample was analysed in triplicate. After PCR was completed, the FAM fluorescent signal (490 nm) was analysed and converted into a relative number of copies of target molecules. These results were expressed by threshold cycle value (Ct = number of necessary amplification cycle that emitted the fluorescent signal superior at non specific fluorescence).

### Immunofluorescence

Cells were incubated with a primary specific antibody. We used the following primary specific antibody for each IP_3_R isoform: rabbit anti-IP_3_R1 polyclonal antibody (1:1000, Affinity Bioreagents), goat anti-IP_3_R2 polyclonal antibody (1:1000, Santa Cruz Biotechnology), mouse anti-IP_3_R3 monoclonal antibody (1:1000, Santa Cruz Biotechnology) and the rabbit anti-calreticulin antibody (1:100, Stressgen Biotechnologies) for 1 h at room temperature. Cells were then incubated with the corresponding conjugated antibody. In the control, the primary antibody was omitted. The nuclei were labelled with TOPRO-3 (1:1000, Interchim). Other details are as described [16].

### Imaging of endoplasmic reticulum

Cells were incubated in 0.5 μM ER tracker (FluoProbes^®^) for 10 min at 37°C. This probe was excited at 488 nm, and the emission (510 nm) was recorded with a spectral confocal station FV 1000 installed on an inverted microscope IX-81 (Olympus Tokyo, Japan).

### Functional assay

Ca^2+^-activated chloride channels activity was assayed on epithelial cell populations by the iodide (^125^I) efflux technique as described [12].

### Recording global calcium signals

Cells were loaded with 3 μM Fluo-4 acetoxymethyl ester (FluoProbes^®^) for 20 min at room temperature and Ca^2+^activity was recorded by confocal laser scanning microscopy using Bio-Rad MRC 1024. All the experiments were performed at minimum on two different cell passages (2 < N < 5), and in each field various cells were selected. This number of cells is noted n on each histogram. Other details are as described [16].

### Monitoring cytosolic Ca^2+ ^transients induced by uncaging Ca^2+^

**Figure 1:**
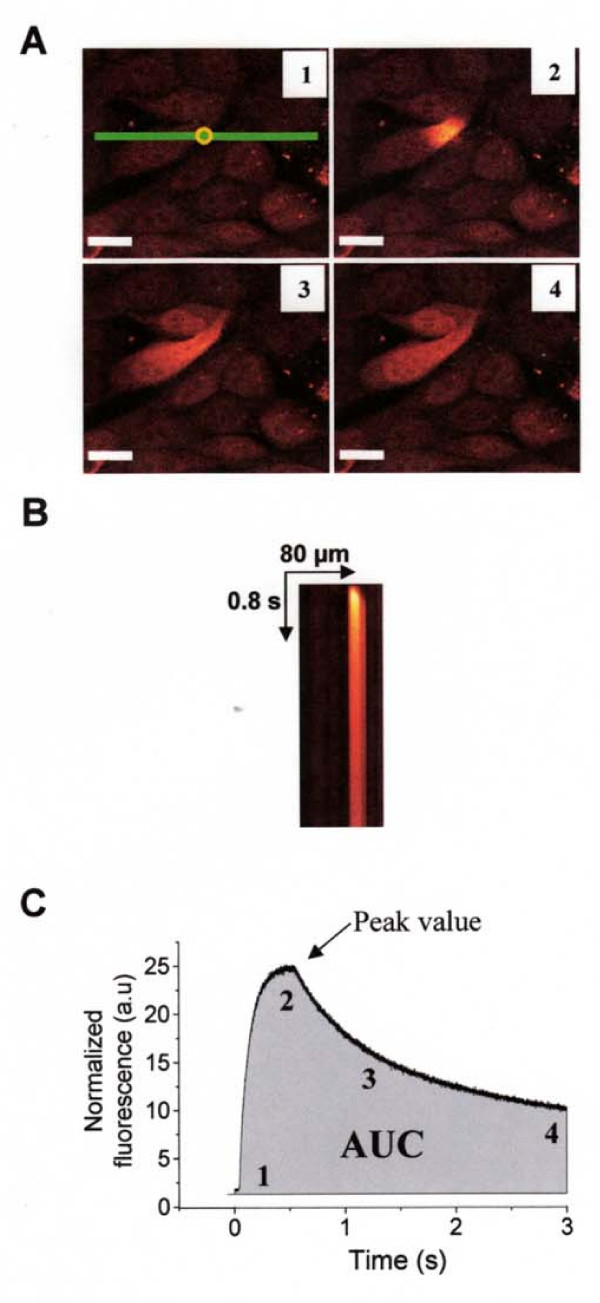
**Determination of localized Ca^2+ ^mobilization by Ca^2+ ^caged technique**. *A *Confocal XY images illustrating Ca^2+ ^release by photolysis of NP-EGTA molecule. The uncaging pulses were delivered with 5% of 405 nm diode for 500 ms with tornado scanning mode in a region of interest of 10 pixels diameter (yellow circle). Scale bars 25 μm. *B *XT images were obtained by acquisition in line scan mode (green line in *A*) during 3 s. *C *Typical intensity profile of Ca^2+ ^variation was extracted from XT images presented in *B*, the grey area represents the measure of area under the curve (AUC). The number 1 to 4 represented the Ca^2+ ^response induce by the photolysis at different time (in figure 1A and 1C). All the parameters automatically measured with a computer program developed in our laboratory under IDL 5.3 structured language were represented on the typical intensity profile (peak and kinetics parameters).

Cells were loaded with 3 μM nitrophenyl-EGTA (NP-EGTA) (Interchim, Montluçon, France) [17] for 40 min, and 20 min with NP-EGTA plus 3 μM Fluo-4 AM at room temperature in buffer solution containing: (in mM) 130 NaCl, 5.4 KCl, 2.5 CaCl_2_, 0.8 MgCl_2_, 5.6 glucose, 10 Hepes, pH 7.4 (adjusted with Tris base). Cells were then washed and allowed to desesterification for 10 min. Ca^2+ ^transients were monitored using confocal laser scanning microscope FV1000 (Olympus, France) installed on an inverted microscope IX-81 (Olympus, Tokyo, Japan) and equipped with two scanning heads. One is used for imaging Fluo-4 fluorescence with 488 nm line of a multi-line argon laser using line scan mode, the other allows stimulation (SIMS) with 405 nm diode. XT images were acquired with ×60/1.2 NA water-immersion objective with 2× optical zoom (spatial resolution of 0.2 μm/pixel) and collected using spectral detector within 500–600 nm. To allow comparison between different experimental conditions, uncaging pulses of the same intensity were delivered with 5% of 405 nm diode for 500 ms with tornado scanning mode in a region of interest of 10 pixels diameter (= 2 μm). Simultaneous scanner system of Olympus FV1000 station allows laser stimulation in a restricted region while recording Fluo-4 fluorescence images with no delay and high resolution. As shown on XY images, laser stimulation with 405 nm diode applied on a restricted region of interest (yellow circle in Fig. 1A) induced a localized Ca^2+ ^increase that propagated throughout the cell. For high time resolution, intracellular Ca^2+ ^images were acquired in a line scan mode during 3 s (XT image, Fig. 1B) with line scan defined in the center of stimulation region (XY reference image, Fig. 1A). 500 ms duration of laser stimulation was chosen for its efficacy to induce large response with no sign of bleach or saturation of cellular response. Typical intensity profile of Ca^2+ ^variation was then extracted from XT images with FV10-ASW v1.3 software within a 10 pixels width region to reduce noise (Fig. 1C). Intensity profiles were normalized by dividing the fluorescence intensity of each pixel (F) by the average resting value before stimulation (F0) to generate an (F-F0/F0) image. With this intensity profile, we compared the different Ca^2+ ^responses by measuring the area under the curve (AUC) and the peak value (Fig. 1C).

### Caged IP_3 _experiments

To activate directly the IP_3_Rs we used the membrane-permeable UV light-sensitive caged IP_3 _analogue, [D-2,3-O-Isopropydylidene-6-O-(2-nitro-4,5-dimethoxy)benzyl-*myo*-inositol 1,4,5-trisphosphate-hexakis(propionoxymethyl)ester] = iso-Ins(1,4,5)P3/PM. Cells were loaded with 1.5 μM iso-Ins(1,4,5)P3/PM (Alexis Biochemicals) [17] for 45 min, and still 20 min with iso-Ins(1,4,5)P3/PM plus 3 μM Fluo-4 AM at room temperature in buffer solution containing: (in mM) 130 NaCl, 5.4 KCl, 2.5 CaCl_2_, 0.8 MgCl_2_, 5.6 glucose, 10 Hepes, pH 7.4 (adjusted with Tris base). Cells were then washed and allowed to desesterification for 20 min. Ca^2+ ^transients were monitored using a confocal laser scanning microscope FV1000 (Olympus, France) in absence of extracellular Ca^2+^. To allow comparison between different experimental conditions, uncaging pulses of the same intensity were delivered with 8% of 405 nm diode for 100 ms with tornado scanning mode in a region of interest of 10 pixels diameter (= 2 μm). Simultaneous scanner system of Olympus FV1000 station allows laser stimulation in a restricted region while recording Fluo-4 fluorescence images with no delay and high resolution. Experiments were conducted at room temperature. Intensity profiles were normalized by dividing the fluorescence intensity of each pixel (F) by the average resting value before stimulation (F0) to generate an (F-F0/F0) image. With this intensity profile, we compared the different Ca^2+ ^responses by measuring the area under the curve (AUC).

### Statistics

Results are expressed as mean ± SEM of n observations. Sets of data were compared with a Student's t test. Differences were considered statistically significant when P < 0.05. ns: non significant difference, * P < 0.05, ** P < 0.01, *** P < 0.001. All statistical tests were performed using GraphPad Prism version 4.0 for Windows (Graphpad Software) and Origin version 5.0.

### Chemicals

2-APB, decavanadate, cyclosporine A, histamine, ATP, A23187 and Caffeine are from Sigma. Thapsigargin is from LC Laboratories. Miglustat and NB-DGJ are from Toronto Research Chemicals.

## Results

### Role of IP_3 _receptors in local ER Ca^2+ ^mobilization in human epithelial cells

**Figure 2:**
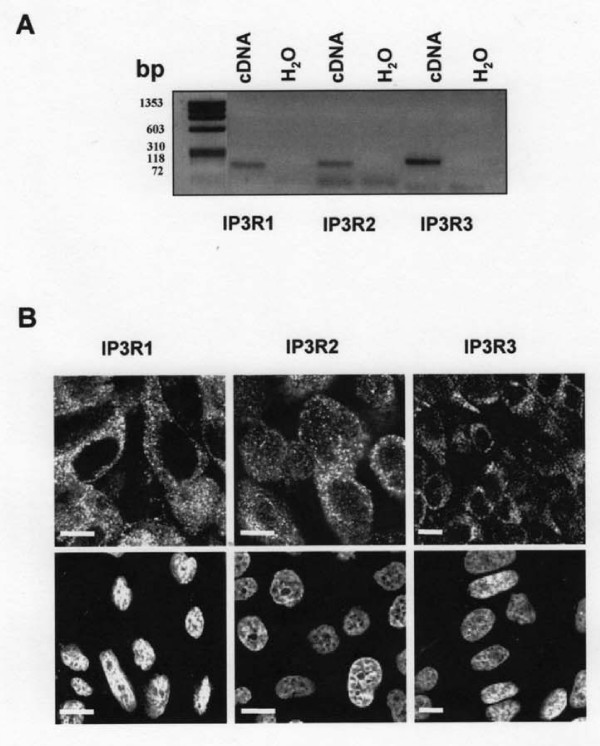
**Characterization of IP_3_Rs isoforms in human nasal epithelial cells**. *A *mRNA amplification of 3 isoforms of IP_3_R by real time PCR. *B *Immunostaining of IP_3_R type 1, 2 and 3 in untreated CF15 cells and staining with the secondary antibody as a negative control (bottom panels); nuclei are labelled with TOPRO-3, bar = 10 μm.

**Figure 3:**
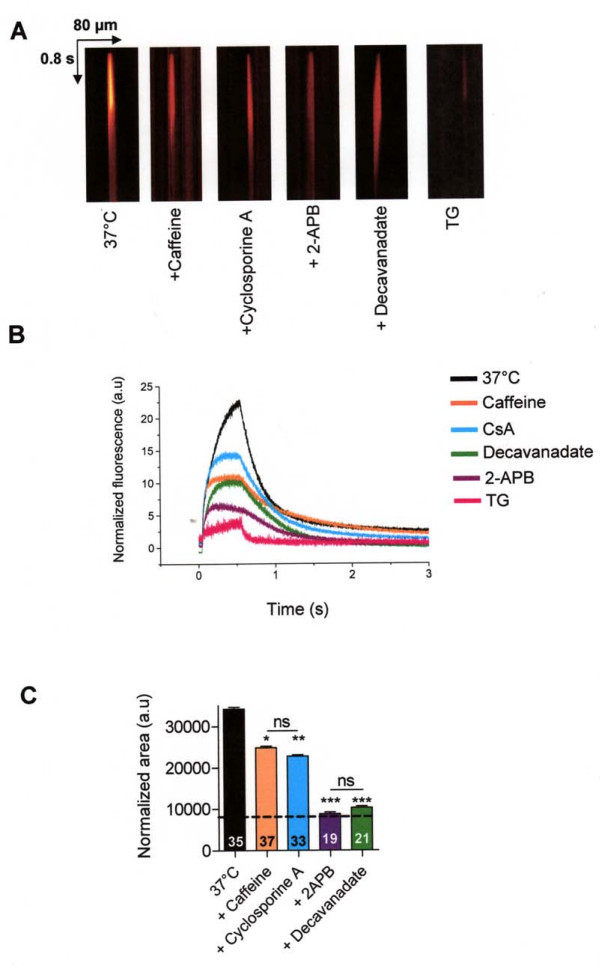
**Pharmacology of IP_3_R response of local uncaging of caged Ca^2+ ^in CF15 cells in absence of extracellular Ca^2+^**. *A *Example of line-scan images acquired at 2 ms per line and 0.21 μm per pixel in CF15 cells untreated at 37°C in presence or not of 100 μM 2-APB, 100 μM decavanadate, 20 mM caffeine or 10 μM cyclosporine A (all were preincubated during 10 min) and after 2 h incubation with 10 μM thapsigargin (TG). *B *Average of the line-scan images in *A *expressed as normalized fluorescence in each conditions *C *Mean normalized area measured from XT images in each experimental condition. The dash line represents the response induced by the flash only, after complete ER Ca^2+ ^store depletion. Results are presented as mean ± SEM and the number of experiments is noted on each bar graph. * P < 0.05; ** P < 0.01*** P < 0.001; ns, non significant difference.

We first characterized IP3R isoforms in human nasal epithelial CF15 cells. Using reverse transcription-PCR technique, we found mRNA for the three isoforms of IP_3_R (Fig. 2A). Moreover, confocal immunofluorescence microscopy studies of IP_3_Rs indicated for each isoform a punctiform and diffuse immunostaining in the cytoplasm of CF15 cells (Fig. 2B top images). No immunostaining of IP_3_Rs was detected when the primary antibodies were omitted (Fig. 2B bottom images). Then, to directly investigate IP_3_R activity, we used the flash photolysis technique to uncage caged Ca^2+ ^into the cytosol of NP-EGTA loaded CF15 cells [17]. Because the capacity of IP_3 _receptors to release Ca^2+ ^into the cytosol is influenced, in part, by the cytosolic local Ca^2+ ^concentration, a confined discharge of Ca^2+ ^by NP-EGTA photolysis induced an activation of Ca^2+ ^release by IP_3 _receptors. To eliminate Ca^2+ ^influx, we performed all experiments in absence of extracellular Ca^2+ ^(Ca^2+^-free). As described in the method section, images were acquired in a line scan mode during 3 s (XT image) with CF15 cells cultured at 37°C (Fig. 3A). The corresponding normalized fluorescence and AUC are shown Fig. 3B (black line) and C (black bar). To study the contribution of IP_3 _receptors into the local Ca^2+ ^release in CF15 cells, we used 2-APB and decavanadate [18], two non specific inhibitors of IP_3_R isoforms. In these experimental conditions, we observed a decrease by more than 70% of the Ca^2+ ^response when we used either 100 μM 2-APB or 100 μM decavanadate (Fig. 3A–C). To prevent the release of Ca^2+ ^by IP_3_Rs from the ER, we measured the response that had been only induced by the flash. We treated cells 2 h with 10 μM thapsigargin (TG) to release the whole Ca^2+ ^store. The light stimulation in presence of TG produced a very small response corresponding only to ~20% of the response obtained at 37°C (Fig. 3A–B). In presence of the receptor blockers, these Ca^2+ ^responses were similar to the response induced only by the photolysis flash (represented in Fig. 3C by a dashed black line). These experiments demonstrate that the total Ca^2+ ^response in human nasal epithelial CF15 cells is due to the activity of IP_3 _receptors.

To discriminate between the different isoforms of IP_3_Rs implicated in Ca^2+ ^release, we used two inhibitors of IP_3_R1 (caffeine, cyclosporine A) in absence of extracellular Ca^2+ ^(Fig. 3). Caffeine is known to inhibit the IP_3_R type 1 and to inhibit this isoform at millimolar concentrations [19]. In our hand, 20 mM caffeine induced an inhibition of Ca^2+ ^response limited to the peak intensity (Fig. 3B). The Ca^2+ ^quantity mobilized in presence of caffeine decreased by 30% (Fig. 3C). We also compared the uncaged Ca^2+ ^response induced by UV flash photolysis in presence of cyclosporine A (CsA), an agent known to abolish type 1 IP_3_R [20]. Cyclosporine A induced a decrease of peak fluorescence intensity and a decrease of Ca^2+ ^quantity mobilization by 45% (Fig. 3B–C). Since, we have shown previously the absence of ryanodine receptors in human nasal epithelial cells line [5], the fraction of Ca^2+ ^response not inhibited by cyclosporine A or caffeine probably arose from the two other isoforms of IP_3_R (type 2 and 3) activity.

### Consequence on local IP3Rs Ca^2+ ^activity of rescuing F508del-CFTR in CF cells

**Figure 4:**
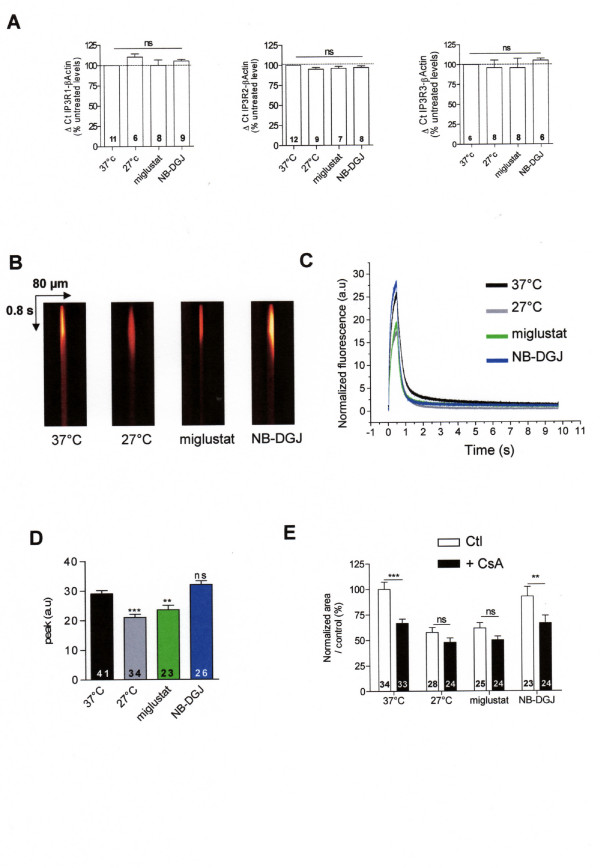
**Modification of local stimulation of caged Ca^2+ ^in corrected F508del-CFTR CF15 cells**. *A *Relative mRNA expression level of IP_3_R-1, IP_3_R-2, and IP_3_R-3 in different conditions compared to βActin mRNA expression. *B *Example of line-scan images acquired at 2 ms per line and 0.21 μm per pixel in CF15 cells treated (27°C, miglustat, NB-DGJ and uncorrected at 37°C in absence of extracellular Ca^2+^). *C *Average of the line-scan images in *B *expressed as normalized fluorescence in absence of extracellular Ca^2+^. *D *Histograms showing the amplitude of IP_3_Rs Ca^2+ ^response in various experimental conditions as indicated. *E *Mean normalized area in each experimental treatment in absence or presence of 10 μM CsA. Sets of data were compared to the control CF15. Results are presented as mean ± SEM and the number of experiments is noted on each bar graph. ** P < 0.01, *** P < 0.001; ns, non significant difference.

To study the consequence of F508del-CFTR rescue on the IP_3_R activity, before loading with NP-EGTA, CF15 cells were either cultured at 27°C during 24 h or incubated 2 h with a culture medium containing 100 μM miglustat. We compared the mRNA quantity of each IP_3_R isoform by quantitative RT-PCR (Fig. 4A), and found no variation of mRNA for each IP_3_R isoforms whatever the experimental conditions (Fig. 4A). The activity of IP_3 _receptors was then evaluated. Example of intracellular Ca^2+ ^XT images are provided for each experimental condition (Fig. 4B). By analysis of the XT images, we observed a decrease by ≈ 40% and 50% in temperature- (24 h at 27°C, grey trace and bar) and miglustat-corrected CF15 cells (2 h at 100 μM, green trace and bar), respectively, compared to uncorrected CF15 cells (37°C, black trace and bar) (Fig. 4C–D). We used NB-DGJ, because this compound is not able to rescue the abnormal trafficking of F508del-CFTR [16]. It is remarkable that treating CF15 cells with NB-DGJ (2 h at 100 μM) did not modify the Ca^2+ ^response compared to untreated CF15 cells as shown by the XT images (Fig. 4B) and the histograms (blue trace and bar Fig. 4C–D). Fig. 4D also provides the corresponding statistical analysis for all these experiments. Therefore, these results show that the rescue of F508del-CFTR either by miglustat or by low temperature deeply affects the capacity of the ER to release Ca^2+ ^into the cytosol of CF15 cells. In each treatment condition, IP_3_R1 inhibition by 10 μM CsA induced a significant decrease of Ca^2+ ^response by 40% in control or NB-DGJ treated cells (Fig. 4E). In contrast, 10 μM CsA did not modify the Ca^2+ ^response in CF15 corrected cells (low temperature or miglustat treatments) (Fig. 4E).

**Figure 5:**
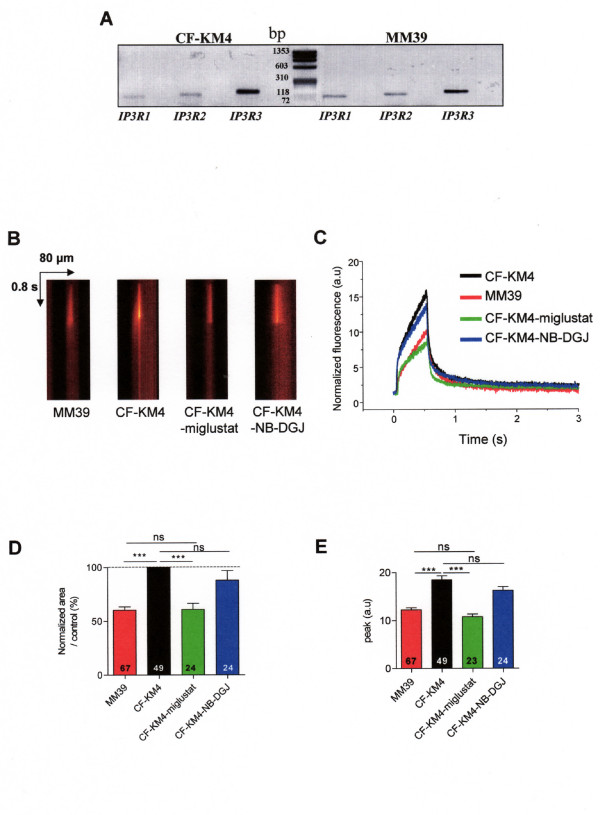
**F508del-CFTR correction in CF-KM4 cells restored histamine ER Ca^2+ ^release compared to non CF MM39 cells**. *A *mRNA amplification of 3 isoforms of IP_3_R by real time PCR in MM39 and CF-KM4 cells. *B *Example of line-scan images acquired in MM39 cells and in uncorrected or corrected CF-KM4 cells in absence of extracellular Ca^2+^. These cells were incubated 2 h at 37°C with 100 μM miglustat or 100 μM NB-DGJ. *C *Average of the line-scan images in *A *expressed as normalized fluorescence in each conditions. *D *Histogram of the normalized area under curve of intensity profile of Ca^2+ ^response extracted from *A *in various experimental conditions as indicated. *E *Mean of amplitude of Ca^2+ ^response in each experimental condition. Results are presented as mean ± SEM and the number of experiments is noted on each bar graph. *** P < 0.001; ns, non significant difference.

To complement this study and to confirm our results, we used two other epithelial cell lines which have another tissue origin: the human tracheal gland serous CF cells (CF-KM4) and non CF cells (MM39). As in CF15 cells, the RT PCR technique shows the presence of IP_3_R1, IP_3_R2 and IP_3_R3 in both human tracheal CF-KM4 and MM39 cells (Fig. 5A). To confirm the exacerbated ER Ca^2+ ^release in CF cells, we also applied the NP-EGTA technique to examined IP_3_R Ca^2+ ^dependent activity in MM39 and CF-KM4 cells. The Ca^2+ ^responses (Fig. 5B–D) showed 40% increase of the Ca^2+ ^response in CF-KM4 cells *versus *non-CF MM39 cells (black and red traces and histograms, respectively). Figure 5B shows line-scan XT images recorded in the absence of extracellular Ca^2+ ^in MM39 cells and in CF-KM4 cells maintained either at 37°C, or at 37°C for 2 h in presence of miglustat or NB-DGJ. The Ca^2+ ^response in CF cells was decreased by ≈ 40% after miglustat treatment (Fig. 5B–E, green traces and histograms). The local Ca^2+ ^response obtained following miglustat treatment was similar to that obtained with the non-CF MM39 cells (Fig. 5C, red traces and histograms). As for CF15 cells, NB-DGJ did not induce any variation of Ca^2+ ^response in CF-KM4 cells compared to uncorrected CF-KM4 cells (Fig. 5D). In fact, the peak of the Ca^2+ ^responses was decreased in non CF MM39 cells and miglustat-corrected CF-KM4 cells compared to uncorrected CF-KM4 cells (Fig. 5C, E). Thus, correction of the abnormal F508del-CFTR trafficking by miglustat induces a profound modification of IP_3_R Ca^2+ ^dependent activity in CF cells.

**Figure 6:**
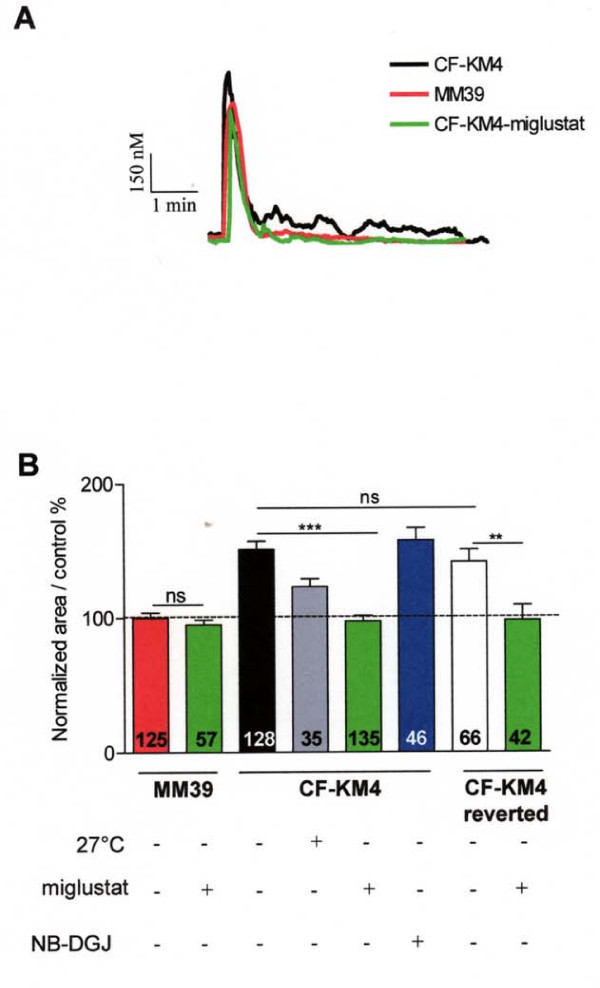
**F508del-CFTR correction in CF-KM4 cells restored local Ca^2+ ^wave propagation compared to non CF MM39 cells**. *A *Typical traces of Ca^2+ ^mobilization in miglustat-treated and untreated CF-KM4 and MM39 during 5 min stimulation by 100 μM histamine in absence of extracellular Ca^2+^. *B *Histogram of the normalized area under the curve corresponding to the cytoplasmic Ca^2+ ^mobilization induced by 100 μM histamine (in 0 mM Ca^2+^) after various treatments. These cells were incubated 2 h at 37°C with 100 μM miglustat (for MM39, CF-KM4 and CF-KM4 reverted cells) or 24 h at 27°C, 100 μM NB-DGJ for CF-KM4 cells. The number on each bar indicates the number of cells. **P < 0.01, *** P < 0.001; ns, non significant difference.

Then, we measured the global ER Ca^2+ ^release (in absence of extracellular Ca^2+^) by 100 μM histamine in control or NB-DGJ treated CF-KM4 or corrected CF-KM4 (by low temperature or miglustat), and on untreated or miglustat-treated MM39 cells (Fig. 6A–B). These experiments show that the Ca^2+ ^response induced by histamine was decreased in CF-KM4 cells corrected either by temperature (by 25%) or miglustat (by 30%), compared to uncorrected CF-KM4 cells (Fig. 6A–B). Example tracings are performed Figure 6A. Again, these ER Ca^2+ ^mobilizations are similar to that observed with MM39 cells. To emphasize the specificity of the effect of miglustat, we also noted that NB-DGJ treatment has no effect on histamine-ER Ca^2+ ^release (Fig. 6B). Furthermore, the histamine-ER Ca^2+ ^release in miglustat-treated MM39 cells was similar to the response observed in untreated MM39 cells (Fig. 6B). Therefore, the decrease of ER Ca^2+ ^release observed in miglustat corrected CF-KM4 cells is not a side effect of miglustat on Ca^2+ ^homeostasis but rather the consequence of F508del-CFTR ER escape.

### Direct activation of the IP_3_Rs using cell-permeable IP3 in human tracheal gland cells

**Figure 7:**
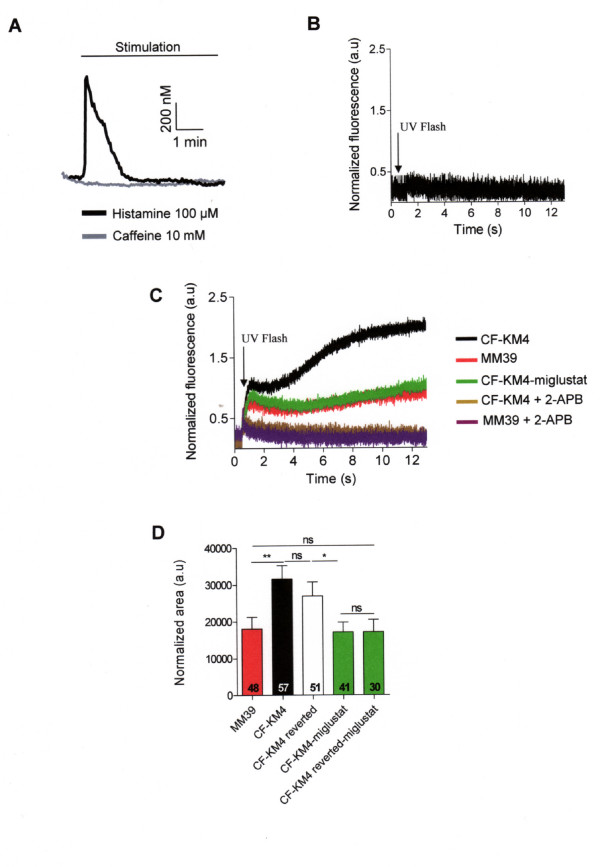
**Flash photolysis of iso-Ins(1,4,5)P3/PM induced release from internal stores in human tracheal gland cells**. *A *Typical traces of Ca^2+ ^mobilization in CF-KM4 cells during 5 min stimulation by 100 μM histamine or 10 mM caffeine in absence of extracellular Ca^2+^. *B *The CF-KM4 cells were loaded with fluo-4 and without iso-Ins(1,4,5)P3/PM and stimulated by UV light. *C *Traces show average normalized fluo4 fluorescence recordings in uncorrected or corrected CF-KM4 (incubated 2 h at 37°C with 100 μM miglustat) and in MM39 cells in absence of extracellular Ca^2+^. These cells were preincubated during 10 min in presence or not of 100 μM 2-APB. *D *Histogram of the normalized area under curve of intensity profile of Ca^2+ ^response in various experimental conditions as indicated. These cells were incubated 2 h at 37°C with 100 μM miglustat (CF-KM4 and CF-KM4 reverted cells). Results are presented as mean ± SEM and the number of experiments is noted on each bar graph. * P < 0.05; ** P < 0.01*** P < 0.001; ns, non significant difference.

Calcium is known to directly activate IP_3_R and ryanodine receptors (RYRs), but the sensitivity of the IP_3 _receptors to Ca^2+ ^depending on a process CICR (Ca^2+ ^increase Ca^2+ ^release) requires IP_3 _[1]. However, in absence of agonist stimulation, the level of intracellular IP_3 _remains very low. When we used 10 mM caffeine to activate specifically RYRs, we did not observe Ca^2+ ^mobilization, on the contrary of 100 μM histamine stimulation (Fig. 7A). These results indicate that RYRs are absent or not functional in these human epithelial cell models (CF15, CF-KM4 and MM39 cells). Then, the Ca^2+ ^response produced by the NP-EGTA photolysis was the consequence of the presence and activity of IP_3_Rs. This explains that the Ca^2+ ^increase observed is only measured during the UV photolysis (500 ms). To eliminate these limitation of the NP-EGTA technique, and to study, more directly, the IP_3_R activity, we examined the ER Ca^2+ ^release by UV light photolysis of a cell-permeable caged iso-Ins(1,4,5)P3/PM in absence of extracellular Ca^2+^. In CF-KM4 cells, preloaded with iso-Ins(1,4,5)P3/PM, short exposure (100 ms) to flash photolysis induced a biphasic increase of [Ca^2+^]_i_. We observed an initial peak of Ca^2+ ^release which stabilized during 1 or 2 s, and an increase of Ca^2+ ^release by a propagation of this Ca^2+ ^response at the whole cell level (Fig. 7C). In MM39 and miglustat-treated CF-KM4 cells, the UV photolysis stimulated a biphasic increase of [Ca^2+^]_i_, but the amplitude of the first peak and Ca^2+ ^mobilization was reduced compared to untreated CF-KM4 cells (Fig. 7C and 7D). Moreover, the second part of Ca^2+ ^response was stabilized, and the rise of Ca^2+ ^release was lower than the response measured for untreated CF-KM4 cells (Fig. 7C). We observed the [Ca^2+^]_i _return to a basal concentration approximately after 15 to 20 s after the UV flash (not shown). To ensure that the response evoked by exposing the cells to UV light was not due to phototoxicity or to a non-specific effect, the experiments were repeated with CF-KM4 cells loaded with fluo-4 without iso-Ins(1,4,5)P3/PM. In this experimental condition, exposure to UV flash did not induce an increase in [Ca^2+^]_i _(Fig. 7B). This experimental procedure confirms that the correction of the abnormal F508del-CFTR trafficking by miglustat induces a profound modification of IP_3_R Ca^2+ ^dependent activity in CF cells.

### Consequence on IP_3_Rs Ca^2+ ^activity of F508del-CFTR ER retention in CF cells

Finally, we used cells derived from CF-KM4 that were stably transfected to achieve low-level expression of full-length wild-type CFTR (wt-CFTR) (CF-KM4-reverted). These CF-KM4-reverted cells have been shown to have phenotypic correction of a wide range of CF phenotypes [14]. In fact, this cell line possesses both CFTR proteins: endogenous F508del-CFTR and transfected wild-type CFTR (wt-CFTR). When we measured the Ca^2+ ^mobilization induced by a solution of 100 μM histamine in absence of extracellular Ca^2+^, this Ca^2+ ^response was also similar to CF-KM4 cells (Fig. 6B). The Ca^2+ ^mobilization induced by UV photolysis of iso-Ins(1,4,5)P3/PM in CF-KM4-reverted was similar to CF-KM4 cells (Fig. 7C). The plasma membrane localization of wt-CFTR did not disrupt the sensitivity of IP_3_Rs to the photolysis of iso-Ins(1,4,5)P3/PM and to agonist response. To restore the endogenous F508del-CFTR trafficking, we treated these cells 2 h at 37°C with 100 μM miglustat. In this condition, the specific IP_3_R activity, measured by Ca^2+ ^response to agonist stimulation and by iso-Ins(1,4,5)P3/PM photolysis was reduced to the level measured in non CF cells (Fig. 6B and 7C). This abnormal increase of IP_3_R Ca^2+ ^release in CF human epithelial cells compared to non CF cells appear thus to be the consequence of F508del-CFTR retention in ER compartment.

### Morphology of the ER in non CF and CF cells

**Figure 8:**
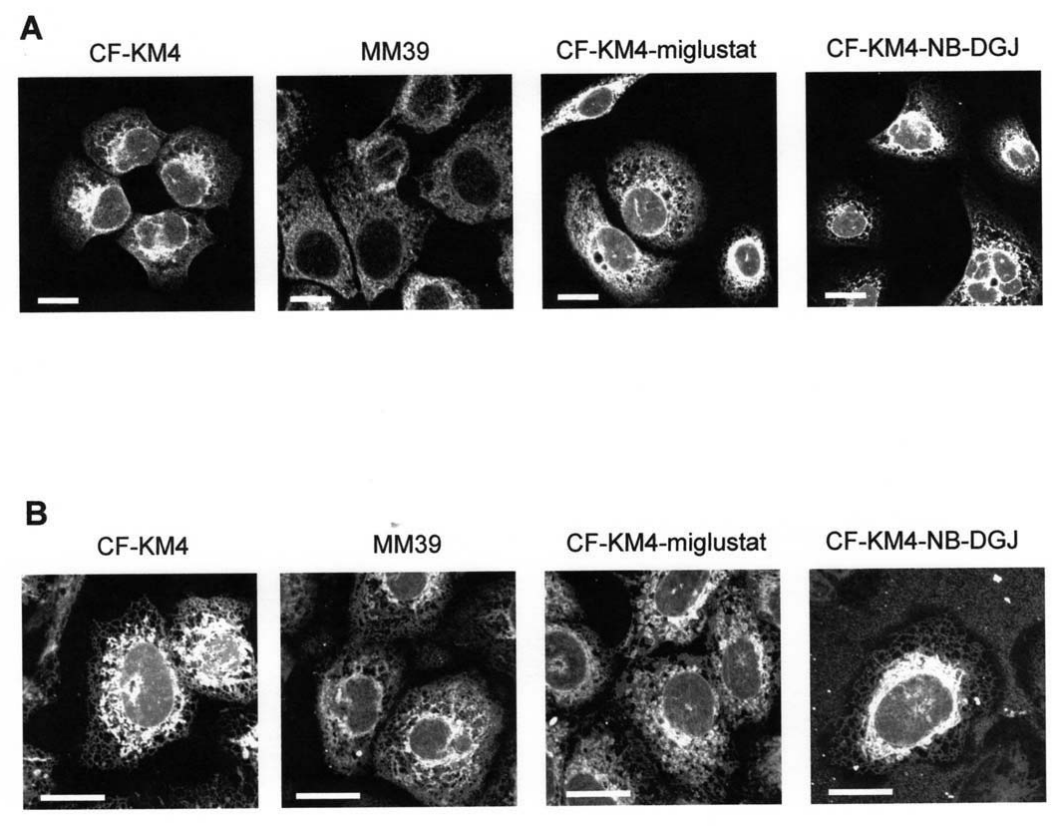
**ER morphology in uncorrected or corrected CF human tracheal gland cells compared to non CF human tracheal gland cells**. *A *Immunostaining of calreticulin in untreated CF-KM4, MM39 and miglustat (100 μM 2 h) or NB-DGJ (100 μM 2 h) treated CF-KM4 cells. Nuclei are labelled with TOPRO-3, bar = 10 μm. *B *ER imaging (with ER tracker probes) in untreated or miglustat (100 μM 2 h) or NB-DGJ (100 μM 2 h) treated CF-KM4 cells and in untreated MM39 cells, bar = 10 μm.

To begin to understand the cause of the ER Ca^2+ ^release abnormal in CF cells, we examined the ER morphology in our experimental conditions. In a first set of experiments, the ER structure was investigated by calreticulin immunofluorescence (Fig. 8A). Calreticulin is an intraluminal ER protein involved in Ca^2+ ^sequestration [21]. Figure 8A shows that the ER remains highly concentrated around the nucleus in untreated or NB-DGJ-treated CF-KM4 cells. On the contrary, in MM39 and in miglustat-corrected-CF-KM4 cells, the ER is spreaded throughout the cells. No immunostaining of calreticulin was detected when the primary antibodies were omitted (data not shown). To verify whether this difference in ER morphology observed between CF and non CF cells is due to the ER structure or to a change of calreticulin localization, we also stained the ER with a specific fluorescent probes (ER tracker) (Fig. 8B). Again, the ER was also found highly concentrated around the nucleus in untreated and NB-DGJ-treated CF-KM4 cells, whereas in periphery of the cells the ER network was very thin. On the contrary, in non CF cells or corrected CF cells, the ER was clearly extended throughout the cell (Fig. 8B). Thus treatment of CF cells with the corrector miglustat induces an ER spreading throughout the cells.

### What is the consequence of the ER Ca^2+ ^decreased on the CaCC activity?

**Figure 9:**
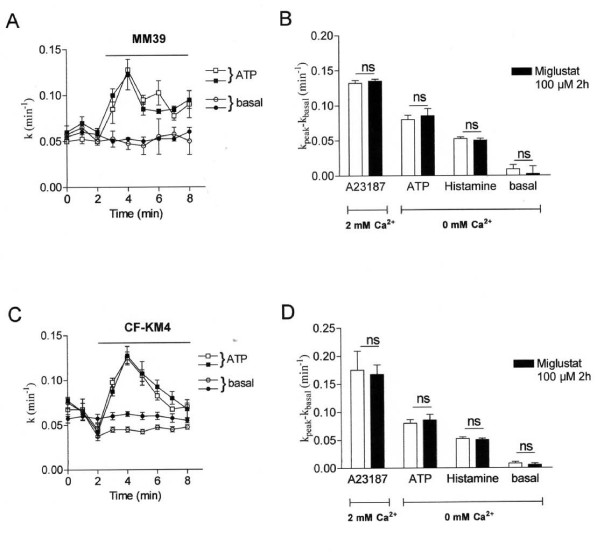
**ER Ca^2+ ^release decreased after F508del-CFTR correction, what is the consequence on calcium-activated chloride channel (CaCC) activity?*** A *Example of mean iodide efflux for activation of CaCC in miglustat-treated (black symbol) or not (open symbol) MM39 cells. CaCC were stimulated by 100 μM ATP in 0 mM Ca^2+ ^bath medium. B Histograms show the mean relative rate for the experimental conditions (1 μM A23187, 100 μM ATP or 100 μM histamine) indicated below each bar (n = 4) in miglustat-treated (black bars) or not (open bars) MM39 cells. *C *Examples of mean iodide efflux for activation of CaCC in miglustat-treated (black symbol) or not (open symbol) CF-KM4 cells. CaCC were stimulated as for MM39 cells. *D *Histograms show the mean relative rate for the experimental conditions indicated below each bar (n = 4) in miglustat-treated (black bars) or not (open bars) CF-KM4 cells. Results are presented as mean ± S.E.M; ns, non significant difference.

Since intracellular Ca^2+ ^regulates the functionality of numerous proteins and because the ER Ca^2+ ^mobilization was decreased in miglustat-CF cells, we determined whether these changes in Ca^2+ ^signalling lead to changes in the Ca^2+ ^mediated Cl^- ^transport. The Ca^2+^-activated Cl^- ^channels (CaCC) are functionally expressed in many non excitable cells [22,23]. We performed iodide efflux experiments in untreated and miglustat-treated MM39 (Fig. 9A) and CF-KM4 (Fig. 9B) cells and stimulated the activity of CaCC by the Ca^2+ ^ionophore A23187. No variation was detected following the treatment of cells by miglustat (Fig. 9). Then we examined the activity of CaCC stimulated by ER Ca^2+ ^release using two different agonists (ATP and histamine). Again no difference was observed between untreated vs miglustat treated cells. Taken altogether, and in spite of the decreased ER Ca^2+ ^mobilization in miglustat-corrected-CF cells, the activity of CaCC remained unaffected by miglustat.

## Discussion

Our study on the regulation of Ca^2+ ^signalling in human F508del-CFTR and in corrected CF cells reveals that (**i**) the release of ER Ca^2+ ^store is dependent on the presence of the three isoforms of IP_3_R, (**ii**) the activity of IP_3_Rs is implicated in the propagation of Ca^2+ ^waves (**iii**) correction of the abnormal trafficking of F508del-CFTR in CF cells regulates local ER Ca^2+ ^release which is correlated to a normalization of this local ER Ca^2+ ^mobilization, (**iv**) IP_3_R1 participation in Ca^2+ ^response is decreased in corrected CF15 cells (**v**) the ER was spreaded throughout the cells, i.e. non CF or corrected CF cells compared to uncorrected CF cells where the ER was condensed around nucleus, (**vi**) the activity of Ca^2+^-dependent Cl^- ^channels are not affected in CF cells, non CF cells, or corrected CF cells.

We propose that Ca^2+ ^homeostasis in cystic fibrosis airway epithelial cells is disturbed and related to the retention in the ER of F508del-CFTR proteins.

Epithelium from trachea to distal intrapulmonary airways (bronchioles) presented positive immunoreactivity for all types of IP_3_Rs [24]. All three isoforms of IP_3_Rs are also expressed in Madin-Darby canine kidney cells, a well studied tight polarized epithelial cell type [25]. Thus, in epithelial cell models, multiple isoforms of IP_3_R appeared to be present in a single cell. In our epithelial models, we showed the presence of the three isoforms. In CF15 cells their localisation is comparable, *i.e*. diffuse in the cytoplasm of the cells. Moreover, no variation of IP_3_Rs mRNA was observed. The three subtypes of IP_3_R Ca^2+ ^release channels share basic properties but differ in term of regulation. Type 1 IP_3_R, with both Ca^2+^-dependent activation and inhibition, is well suited for establishing Ca^2+ ^oscillations [1,26,27], where the frequency of Ca^2+ ^transients can be modulated when IP_3 _concentrations are increased [27,28]. The effects of CsA are lower in CF15 corrected cells than in uncorrected CF15 cells; it suggests that the CsA-sensitive IP_3_R participation in Ca^2+ ^response was decreased in CF15 corrected cells.

Human CF primary bronchial epithelial cells and respiratory cell lines were reported to produce an exaggerated proinflammatory cytokine response associated with an activation of NF-κB [29-31]. Intracellular Ca^2+ ^is known to play a central role in production and secretion of Il-8 [32,33]. The IL-1β stimulation induces a prolonged [Ca^2+^]_i _in IB3-1 cells which was correlated to NF-κB activation [34]. The deregulation of IP_3_R Ca^2+ ^release observed in human nasal and tracheal epithelial cells could be implicated in increasing inflammatory response observed in numerous CF cell lines in particular in CF epithelial cells [6,34].

The apical ER network is expended in human CF bronchial epithelial cells compared to ER volume in human non CF bronchial epithelial cells [6]. In this present study, the ER staining (by calreticulin immunostainning or ER tracker probe) shows that the ER structure is highly different in CF compared to non CF or CF-corrected cells. The ER volume seems to be concentrated around the nucleus in CF cells and expanded throughout the cytoplasm of non CF and CF-corrected cells. This expansion could be responsible for the variation in IP_3_R Ca^2+ ^dependent activity observed in this present study. Indeed, the display of ER web could induce probably an augmentation of distance between IP_3 _receptors which would induce a decrease in the propagation of the Ca^2+ ^response. Furthermore, the F508del-CFTR correction is causing a potential redistribution of IP_3_Rs at the ER membrane. We believed that in corrected CF and non CF cells, IP_3_Rs are more distant from each others, leading to reduce propagation of the Ca^2+ ^wave. Then IP_3_Rs clustering of the ER could facilitate the formation of highly sensitive Ca^2+ ^release sites, thereby stimulating the Ca^2+ ^wave initiation and propagation [35]. For example, in maturation of oocytes, the modifications in ER clusters are accompanied by an increase in the sensitivity of Ca^2+ ^release by IP_3 _[36,37]. Moreover, in CF cells IP_3_Rs are closer to each other and the photolysis induces a long Ca^2+ ^wave due to a better propagation. Our interpretation of the data obtained with CF-KM4-reverted cells is that the IP_3_Rs deregulation is not due to the CFTR absence at the plasma membrane but is more likely due to the abnormal ER F508del-CFTR retention. The F508del-CFTR escape of ER by pharmacological corrector treatment induces the normalization of IP_3_R Ca^2+ ^release.

In the airways, pleiotropic consequences accompanied the production of F508del-CFTR protein generating vicious cycle of airway obstruction, infection, and inflammation at the origin of most of the morbidity and mortality in cystic fibrosis. The pro-inflammatory mediator bradykinin triggers Ca^2+ ^mobilization [38,39] and induces interleukin-8 secretion in non-CF and CF human airway epithelia [40,41]. A mechanism has been proposed to explain the increase of Ca^2+ ^signals at the apical membrane in human CF airway epithelia; it results from the expansion of the ER Ca^2+ ^store compartment rather than from a greater number of purinergic receptors [6,40]. We can confirm these results in our cellular models because we observed that the kinetics of activation of the Ca^2+ ^response obtained in NB-DNJ-treated CF cells do not mimic the effect of the inhibitors of the IP_3_R and we observed a variation of the ER morphology between non CF or corrected and uncorrected epithelial cells.

We recently provided evidence that the pharmacological correction of abnormal trafficking of F508del-CFTR [12] induces a restoration of Ca^2+ ^mobilization in CF cells [5]. Here, we showed reduction of the inositol 1,4,5-trisphosphate receptors (IP_3_R) dependent-Ca^2+ ^response following two different correcting treatments compared to uncorrected cells in such a way that Ca^2+ ^responses (CF+treatment *vs *wild-type cells) were normalized. Altogether, these results suggest reversal of the IP_3_R dysfunction in F508del-CFTR epithelial cells by a pharmacological correction of the abnormal trafficking of F508del-CFTR in cystic fibrosis cells.

## Abbreviations list

AM: Acetoxymethyl; 2-APB: 2-aminoethyoxydiphenyl borate; CF: cystic fibrosis; CFTR: cystic fibrosis transmembrane conductance regulator; ER: endoplasmic reticulum; IP_3_: inositol 1,4,5-trisphosphate; IP_3 _caged: iso-Ins(1,4,5)P3/PM; IP_3_R: IP_3 _receptor; NB-DGJ: N-butyldeoxygalactojirimycin; NB-DNJ: N-butyldeoxynojirimycin; NP-EGTA: Nitrophenyl-Ethylene Glycol-bis(β-Aminoethyl ether) N,N,N',N'-Tetraacetic Acid; TG: Thapsigargin; CsA: Cyclosporine A.

## Competing interests

The authors declare that they have no competing interests.

## Authors' contributions

FA conducted the experiments, analysed the data and wrote the draft of the manuscript. CN realized the iodide efflux experiments. AC helped us to realized confocal microscopy experiments and analysis. FB and CV revised the manuscript. All authors read and approved of the final manuscript.
